# Effect of acceleration of auditory inputs on the primary somatosensory cortex in humans

**DOI:** 10.1038/s41598-018-31319-3

**Published:** 2018-08-27

**Authors:** Shunsuke Sugiyama, Nobuyuki Takeuchi, Koji Inui, Makoto Nishihara, Toshiki Shioiri

**Affiliations:** 1 Department of Psychiatry and Psychotherapy, Gifu University Graduate School of Medicine, Gifu, Japan; 2 Depatment of Psychiatry, Aichi Medical University, Nagakute, Japan; 3 Institute for Developmental Research, Aichi Human Service Center, Kasugai, Japan; 4 Department of Integrative Physiology, National Institute for Physiological Sciences, Okazaki, Japan; 5 Multidisciplinary Pain Center, Aichi Medical University, Nagakute, Japan

## Abstract

Cross-modal interaction occurs during the early stages of processing in the sensory cortex; however, its effect on neuronal activity speed remains unclear. We used magnetoencephalography to investigate whether auditory stimulation influences the initial cortical activity in the primary somatosensory cortex. A 25-ms pure tone was randomly presented to the left or right side of healthy volunteers at 1000 ms when electrical pulses were applied to the left or right median nerve at 20 Hz for 1500 ms because we did not observe any cross-modal effect elicited by a single pulse. The latency of N20 m originating from Brodmann’s area 3b was measured for each pulse. The auditory stimulation significantly shortened the N20 m latency at 1050 and 1100 ms. This reduction in N20 m latency was identical for the ipsilateral and contralateral sounds for both latency points. Therefore, somatosensory–auditory interaction, such as input to the area 3b from the thalamus, occurred during the early stages of synaptic transmission. Auditory information that converged on the somatosensory system was considered to have arisen from the early stages of the feedforward pathway. Acceleration of information processing through the cross-modal interaction seemed to be partly due to faster processing in the sensory cortex.

## Introduction

Humans receive several multisensory signals including, visual, audible, and tactile stimuli. Traditional theories demonstrate that the initial processing of multisensory signals occurs independently for each modality, with integration occurring during the later processing stages^1,2^. However, recent studies on multisensory integration have proved that multisensory processing occurs in early stages^3–6^. Human and animal studies have shown that multisensory interactions can occur in brain regions that were once considered unisensory^7^. Multisensory interaction has been reported more in the auditory cortex than in other sensory systems^8–21^. Studies using functional magnetic resonance imaging (fMRI)^8–11^, event-related potentials (ERPs)^12–16^, and magnetoencephalography (MEG)^17,18^ have showed that visual and somatosensory interactions occur in the human auditory cortex. Intracranial recordings in macaques have directly confirmed audio-visual and audio-tactile convergence in the sub-regions of the auditory cortex^19,20^. In addition, neurons in the primary auditory cortex respond to both auditory and somatosensory stimuli in macaques^21^. Regarding multisensory interaction in the visual cortex, Morrell^22^, in his early unitary recording study on awake cats, has shown that neurons in the visual cortex can be driven by auditory stimuli. Furthermore, studies on monkeys have shown audio-visual convergence in the visual cortex^23,24^. In humans, fMRI and transcranial magnetic stimulation studies have revealed multisensory interactions in the visual cortex^25–27^.

These findings suggest that the convergence of sensory information from different modalities occurs during the early stages of sensory processing. To date, however, whether signals from other sensory systems modulate the initial stage of hierarchical processing in the somatosensory cortex in humans remains unclear. Although studies on visual and somatosensory processing in the auditory cortex and on auditory and somatosensory inputs to the visual cortex have been conducted, only few have examined visual or auditory interactions in the somatosensory cortex. An anatomical tracer study showed that visuo-somatosensory projections originate from the visual cortex in monkeys^28^. However, only a few studies have reported on projections to the somatosensory cortex originating from other sensory cortices^29^. When monkeys perform a haptic task, neurons in the somatosensory cortex are activated by auditory stimuli^30^. Furthermore, recent fMRI studies on humans have suggested responses to auditory and visual stimulation in classically defined somatosensory areas^31–33^. Studies using cross-modal mismatch negativity paradigms have reported that auditory and somatosensory interactions take place in unisensory areas in humans^34,35^. Studies on neuronal oscillations have shown that neurophysiological mechanisms underlie the early multisensory interaction^36–41^. Phase alignment has also been shown to play a vital role in inter-regional communication. Current source density (CSD) studies have reported an explicit cross-modal interaction in unisensory areas in macaques, such that a sensory stimulus modulates the activity in the primary sensory cortex of another sensory system by resetting the phase of ongoing oscillatory activity^37–39^. With respect to audio-visual interaction, such a mechanism was also shown to exist in humans using electroencephalography and transcranial magnetic stimulation^40^. It is noteworthy that some of these studies, using neural oscillations, reported an acceleration of the cross-modal interaction. Mercier *et al*.^41^ recorded electrocorticograms and reaction times in patients with epilepsy when auditory, visual, or audio-visual stimuli were simultaneously presented. The study found that higher synchronization in the auditory area results in faster response time, suggesting a vital role of the cross-modal interaction in the multisensory facilitation of reaction times. In addition, a CSD study on monkeys reported a correlation between the phase of delta oscillation and reaction time^38^.

We aimed to check whether the initial cortical activity of the primary somatosensory cortex (S1) is influenced by a different sensory system. We recorded N20 m following median nerve (MN) stimulation with MEG and examined the effects of a simultaneously presented sound on its latency. The somatosensory system showed a considerable advantage over the auditory and visual systems in the present study because the initial cortical activity can be easily and clearly observed even during repetitive stimulations^42–44^. MEG can record brain activity in the millisecond range; this is useful for investigating the effects of auditory stimulation on the flow of cortical processing. We hypothesized that auditory stimulation shortens the latency of N20 m due to the accelerative nature of cross-modal interaction^45^.

## Results

**Figure 1:**
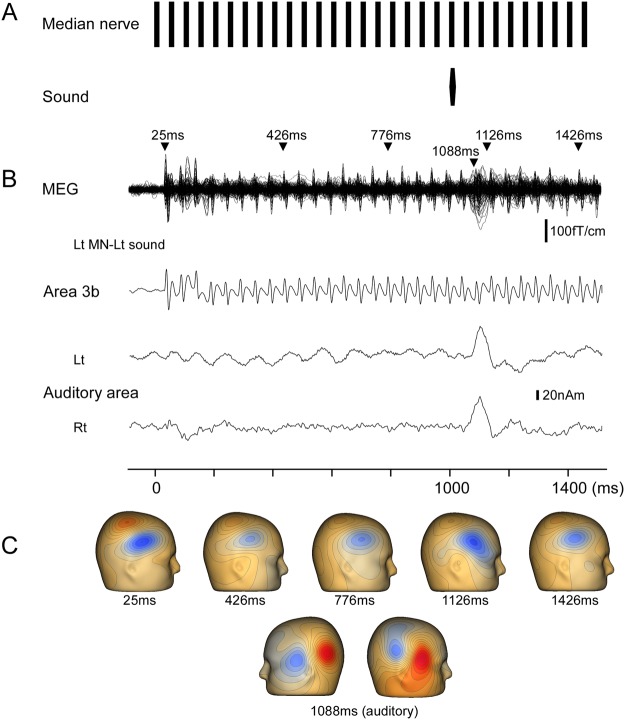
Effects of sound on N20 m following median nerve stimulation. The figure shows N20 m data for a representative participant following left median nerve (MN) stimulation with a pure tone to the left ear. **(A)** Stimulation paradigm. MN stimulation was achieved with a train of current-constant square-wave pulses at 20 Hz. A pure tone of 25-ms duration was presented at 1000 ms. **(B)** Superimposed MEG waveforms recorded using 204 gradiometers, and source-strength waveforms of dipoles in area 3b and left auditory cortex. **(C)** Three-dimensional maps of magnetic fields recorded from gradiometers. The field distribution was similar at each N20 m peak on visual inspection. Maps are shown at several N20 m peaks as examples to avoid redundancy. Latencies of the upper panel show the peak latencies of N20 m and that of the lower panel show the peak latency of auditory N100 m.

**Figure 2:**
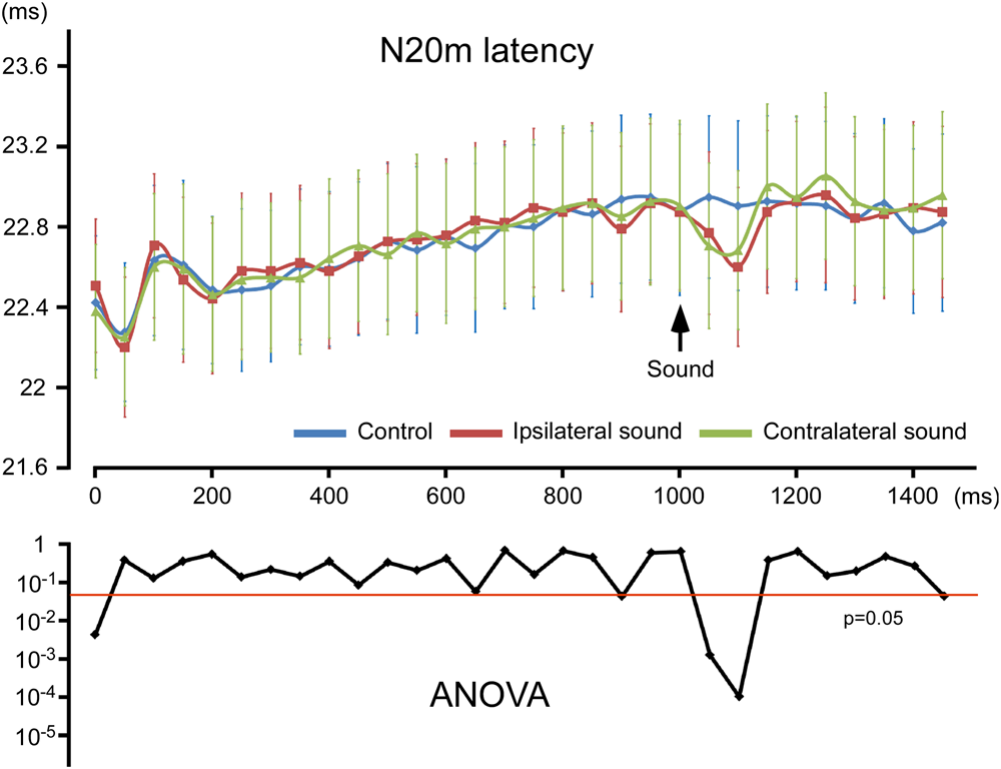
Peak latency of N20 m for each pulse. The mean peak latency of N20 m for each stimulus (top) and p values for the effect of sound obtained using two-way ANOVA (bottom). Vertical bars, ±1 standard error.

MN stimulation induced a clear early component, which peaked at approximately 23 ms (N20 m). The original MEG and source-strength waveforms for S1 belonging to one participant are shown in Fig. 1 as an example. The N20 m latencies for each condition are listed in Supplementary Table 1. As shown in Fig. 2, the N20 m latency became longer with repetitive stimulations, reaching approximately 800 ms after the onset of stimulation, with the latency at 1000 ms being longer than that at 0 ms by 0.46 ms on average (p = 9.52 × 10^−4^; paired *t*-test) in the two control conditions with left or right MN only. In the two control conditions with the left or right MN only, the N20 m latencies within the range of 0–700 ms were significantly shorter than those at 1000 ms (p < 4.28 × 10^−2^; paired *t*-test), whereas those within the range of 750–950 ms did not significantly differ from those at 1000 ms (p > 5.74 × 10^−2^). Here “1000 ms” indicates the N20 m response following the electrical pulse delivered at 1000 ms.

**Figure 3:**
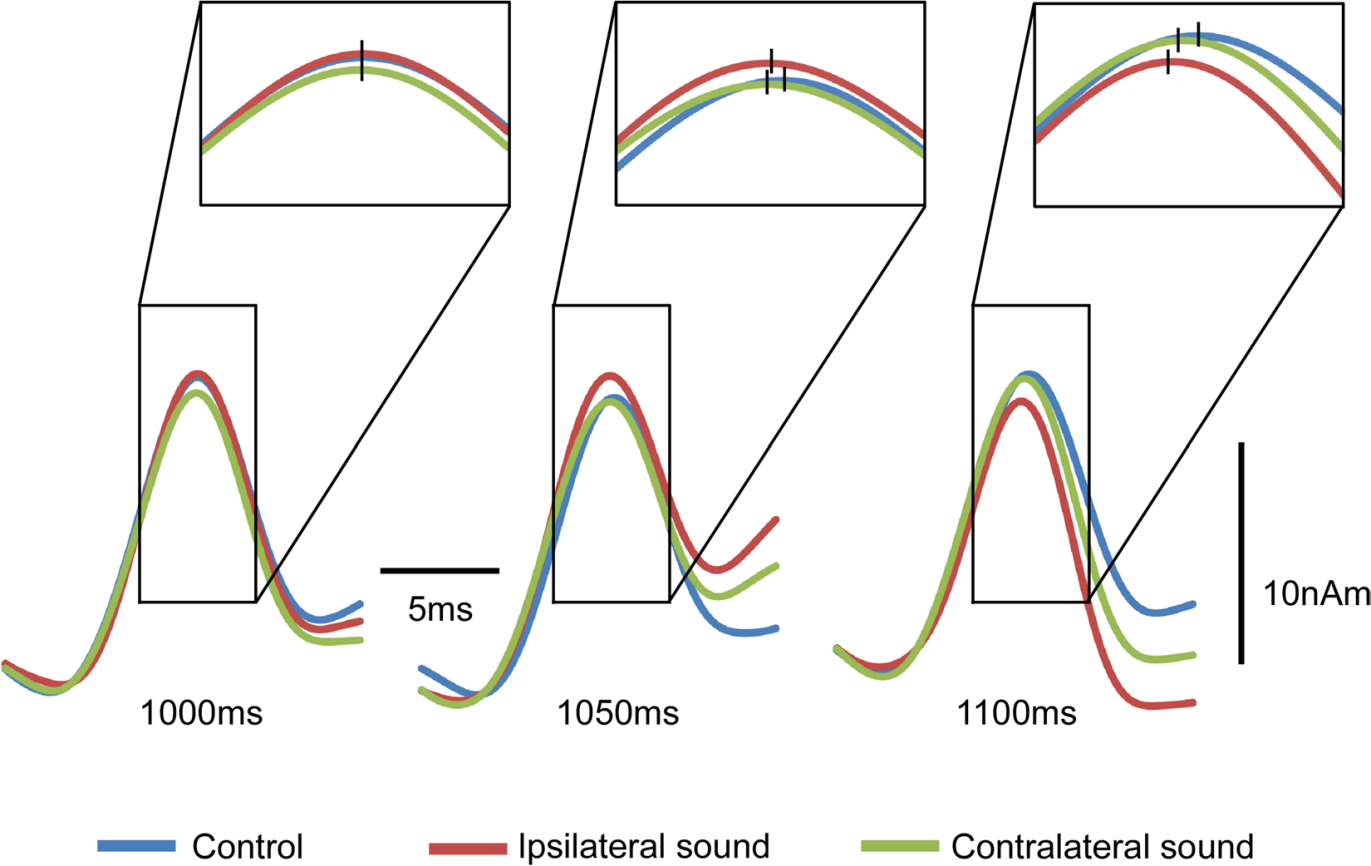
Acceleration of audio-somatosensory interaction with respect to N20 m. Grand-averaged waveforms of N20 m for 12 participants at 1000, 1050, and 1100 ms. Vertical bars, the mean peak latency of N20 m for the conditions.

Figure 3 shows the grand-averaged waveforms of N20 m at 1000, 1050, and 1100 ms. The results of two-way analysis of variance (ANOVA; hemisphere × sound) revealed that sound significantly affected the N20 m latency at 1050 ms (F_2, 46_ = 9.93; p = 7.79 × 10^−4^) and 1100 ms (F_2, 46_ = 15.85; p = 4.71 × 10^−5^) (Fig. 2). The N20 m latencies showed a normal distribution at all sampling points (p < 0.05), according to the Shapiro–Wilk test. Post-hoc tests revealed that both ipsilateral (p = 0.003) and contralateral (p = 0.009) sounds significantly shortened the N20 m latency at 1100 and 1050 ms (0.041 and 0.006). No difference was observed between the ipsilateral and contralateral sounds at 1100 or 1050 ms. Brain hemisphere did not play a role in determining the N20 m latency in any condition (p > 0.22). On average, the N20 m latency with sound was shorter than that without it by 0.21 ms at 1050 ms and by 0.27 ms at 1100 ms. Although the difference between stimuli conditions for the first pulse was significant (Fig. 2), we could not determine an appropriate explanation for this. At this point, the marked difference was caused by a slightly longer latency for the MN + contralateral sound condition compared with MN (p = 0.50), with a slightly shorter latency for the MN + ipsilateral sound condition (p = 0.12). Because no sound was present at this point, it was assumed to be an artifact.

To evaluate the influence of anticipation effects, all data were divided in half and the effect of the sound for four conditions (left and right MN × left and right sound) at 1050 and 1100 ms was compared with the first and second halves of the main experiment. The *t*-tests did not reveal any significant difference for all the conditions at 1050 and 1100 ms (p > 0.09; paired *t*-test uncorrected for multiple comparisons; Supplementary Fig. 1), which is consistent with the results of Preliminary Experiment 3 described in Methods section.

## Discussion

This study examined the effects of sounds on the N20 m latency to elucidate the cross-modal interaction in the somatosensory cortex. Our results revealed that the sound presented before the MN stimulation markedly shortened the N20 m latency, which is congruent with the idea that the cross-modal interaction shortens physiological reactions to sensory stimuli. Because the N20 m component of a magnetoencephalogram reflects the excitatory post-synaptic potentials of inputs from the thalamus to the first cortical area, Brodmann’s area 3b of the somatosensory system^46–48^, interaction occurred at the early stages of somatosensory processing. In addition, information arising during the early stages of the auditory processing seems accountable for the interaction because sound influenced the N20 m latency with a short conditioning-test interval of 50–100 ms. However, several reasons can be attributable for the site of convergence and pathway for the auditory information to reach the interaction site.

### Site of convergence

Because the N20 component reflects synaptic events in area 3b^46–48^, the interaction might occur during the synaptic transmission from the thalamus to area 3b or during earlier stages. This notion is supported by single-unit recording studies on monkeys showing that low-level sensory areas receive inputs from other sensory modalities^7^. First, the possibility that area 3b is the target convergence site is supported by studies reporting on heteromodal connections among low-level cortical regions in monkeys^23,24,28,49^. Regarding the input from the auditory to the somatosensory cortex, direct and reciprocal connections between the primary auditory cortex (A1) and S1 have been shown in Mongolian gerbils^29,50^. A second possible convergence site is the superior colliculus (SC), which is also a site of cross-modal interaction^51^. However, we could not find a spatial effect of sound on the N20 m latency, which is not in accordance with the spatial importance of integration in SC indicated by its topographical organization^51^. The third possible integration site is the thalamus. Signals from the sensory system reach the primary sensory cortex of a different modality via the thalamocortical pathway. A study on gerbils has shown that 5% of the thalamic inputs to S1 come from non-somatosensory nuclei, including the medial geniculate body^50^. In addition to the feedback projections, the thalamus receives feedforward projections from the sensory cortex and, in turn, sends the information to another sensory cortex. Therefore, the thalamus can connect distant cortical regions via a cortico–thalamo–cortical pathway and therefore may contain multisensory information. Such feedforward projections have been demonstrated in several sensory and motor areas^52^.

### Origin of auditory information inputs to the somatosensory system

Sound presented 50 ms before the MN stimulation significantly shortened the latency of N20 m. Therefore, the auditory information that converged on the somatosensory system may have arisen during the early processing stages. We have previously shown that the auditory information takes 30–50 ms from a click tone to reach the higher auditory areas around the superior temporal gyrus^53^. The time taken to reach the cortex should have been slightly higher in the present study because we used a pure tone of 25 ms. It is possible that the early stages of the auditory cortex are the origin sites rather than multimodal areas. Therefore, the cortico–thalamo–cortical pathway is not a likely candidate because there is little evidence that S1 participates in this circuit^52^. Given that cross-modal interaction in SC depends on functional inputs from multimodal association areas^54^, it is also an unlikely candidate as the origin site. Furthermore, the importance of the feedback pathway from association areas for cross-modal interaction has been validated^20,54–59^. Because various stages of sensory processing in one sensory modality receive nonspecific inputs from other modalities^5,7^, our results suggested that there is at least one interaction mechanism that does not require the feedback pathway. This is supported by a study using CSD analysis in monkeys^19^. Studies on monkeys have also revealed that corticocortical information transfer follows the feedforward-type laminar organization of multimodal connections between low-level sensory areas^28^. As for connections from A1 to S1, a laminar projection pattern has been shown to exist in gerbils that suggests a feedforward projection^29^.

Our findings on multimodal interaction during the early stage of sensory processing are consistent with those of previous ERP studies. A study investigating the time course of multisensory interactions between simultaneous auditory and somatosensory stimulations found significant interactions in evoked potentials at an onset latency of 50 ms^13^. Interestingly, a follow-up ERP study by the same group demonstrated that this early interaction was not affected by the spatial alignment of the two stimuli^60^, which is consistent with our results. This suggested that the early interaction does not depend on the relative location of sensory information. Besides audio-somatosensory interactions, early interactions in ERPs have been shown to exist between the auditory and visual systems^12,14,61,62^. Taken together, the direct corticocortical or thalamocortical projection to S1 from the early stage of the auditory pathway appears to be a likely candidate for the present multisensory interaction, as discussed by Henschke *et al*.^50^.

### Functional implications

In addition to improving the detection threshold and accuracy^63–66^, reaction times are often used to evaluate the effects of cross-modal interaction^67^. Reaction times to two simultaneous stimuli are faster than reactions to either of them presented on their own^68^. Studies using reaction times to investigate visual and auditory interaction have been published since then^69–72^. Despite evidence showing that cross-modal interaction shortens physiological reactions to sensory stimuli, the underlying mechanisms remain largely unknown. Our current results indicated that faster processing in S1 could shorten reaction times. The study by Sperdin *et al*.^73^ supports this idea, showing that the initial interactions between neural responses were directly related to reaction times. However, the shortened time of roughly 0.25 ms observed in this study was much lower than that reported by psychophysical studies; the reaction time to a tactile stimulus with a simultaneous sound has been confirmed to be 10–20 ms faster than that to a stimulus without sound^68^. Moreover, the reaction time to audio-tactile stimulation is approximately 25 ms shorter than that to unimodal stimulation^60^. Although multimodal effects on the response latency in the somatosensory pathway have not been investigated, other relevant studies do exist. Wang *et al*.^74^ investigated the effects of visuo-auditory interactions on V1 neurons in awake monkeys performing a saccade task and found that for a visual stimulus with a medium contrast level, simultaneous auditory stimulation reduced the response latency of V1 neurons by 3.5 ms (61.0 vs. 64.5 ms) and the saccade reaction time by 10–15%. Visuo-auditory stimulation reduced the response latency of SC neurons by approximately 10 ms in awake monkeys^75^ and by 6.2 ms in anesthetized cats^45^. Bell *et al*.^75^ have also demonstrated that multisensory interactions influence premotor activities in SC. Modulation of the response latency by multisensory stimulation was also shown in an association cortex in awake monkeys^76^. Although how brain area contributes to the acceleration of the ultimate motor reaction remains unclear, it is conceivable that the sensory, multimodal, and motor areas contribute by both augmenting and quickening the responses. The shortening effect may be shorter for SC than that for the sensory cortex because SC requires functional inputs from the association cortex for cross-modal interaction^54^. If there are multisensory interactions at each stage of the hierarchical sensory processing and motor execution, then the reduction in the final response time must reflect the cumulative effects. Therefore, we believe that a reduction of 0.25 ms is a small, but functionally significant, effect.

In Preliminary Experiment 1 described below, we could not find any latency effect of sound when N20 m was elicited by a single pulse. Although modulating effects of the sound were present, it is likely that there was no room to be affected by the sound. Increasing the stimulus intensity does not considerably affect the N20 latency following MN stimulation^77^, i.e., the N20 m latency following a single pulse would reach a limit in this study. Bell *et al*.^75^ showed that the latency shortening effect of audio-visual stimulation on V1 neurons was negatively correlated with the response latency to visual stimulation alone, suggesting that cells with longer latencies show a greater reduction. This may be because the effects of multisensory stimulation are greater when the baseline response of a neuron has more potential to be modulated. Such a mechanism could contribute to synchronized firing and, thereby, to augmented and faster responses.

## Conclusions

The rapid processing of sensory information is necessary for animals to survive and is considered a basic objective of multisensory integration. To the best of our knowledge, the present study is the first to report that audio-tactile cross-modal interaction shortens the initial cortical activity in the human somatosensory cortex, indicating that cross-modal interaction takes place during the initial stage of cortical processing and that faster processing in the sensory cortex could contribute to shorter reaction times under multisensory integration.

## Methods

This study was approved in advance by the Ethics Committee of the National Institute for Physiological Sciences, Okazaki, Japan, and was conducted in accordance with the Declaration of Helsinki. Written informed consent was obtained from all participants. We enrolled 12 healthy volunteers (3 women and 9 men) aged 27–54 years (35.8 ± 8.9 years). None of the participants had any history of mental or neurological disorders or substance abuse in the last 2 years, and were free of medication at the time of testing. They had a hearing threshold of <30 dB at 1000 Hz as assessed using an audiometer (AA-71, Rion, Tokyo, Japan).

### Somatosensory and auditory stimulation

Somatosensory-evoked magnetic fields were elicited using a train of current-constant square-wave pulses 0.2 ms in duration at 20 Hz, applied to the left and right MN at the wrist using a felt-tip bipolar electrode. The participants were stimulated on both the left and right wrists in separate trials. The intensity of the stimuli was the threshold for thumb twitching. The stimulus involved 30 pulses with a total duration of 1500 ms (Fig. 1A).

A pure tone 25 ms in duration (rise/fall, 5 ms) and 90-dB SPL sound pressure were simultaneously presented to the left and right side with the 21^st^ MN stimulation at 1000 ms of the pulse train. The sound stimulus was presented via earpieces (E-A-Rtone 3 A, Aero Company, Indianapolis, IN). The sound pressure was controlled using an audiometer (AA-71, Rion, Tokyo, Japan). The timing of the sound delivery was controlled using our own proprietary software.

### MEG recordings

Magnetic signals were recorded using a 306-channel whole-head MEG system (Vector-view, ELEKTA Neuromag, Helsinki, Finland) comprising 102 identical triple sensor elements. Each sensor element comprised two orthogonal planar gradiometers and one magnetometer coupled with a multi-superconducting quantum interference device, which provided three independent measurements of the magnetic fields. We analyzed MEG signals recorded from 204 planar-type gradiometers, which were sufficiently powerful to detect the largest signal only over local cerebral sources. Signals were recorded with a bandpass filter of 0.1–300 Hz and were digitized at 4000 Hz. Epochs with MEG signals of >2.7 pT/cm were excluded from the average values. The waveform was digitally filtered with a bandpass filter of 1–200 Hz and a notch filter of 17.5–22.5 Hz. Because we focused only on N20 m, the notch filter was used to exclude the 20-Hz steady state responses. Multisensory effects on steady state response are known to occur in the auditory and visual systems^78–82^. However, we found that the filter had no effect on the N20 m latency, including at 1050 and 1100 ms when the sound showed a significant acceleration. For example, in the left MN + left sound condition in the Preliminary Experiment 2, the N20 m latency was almost identical to that obtained without the notch filter at each pulse (Supplementary Fig. 2); the mean difference for 30 pulses between filter settings was 0.0011–0.0069 ms (for four participants).

### Procedure

The experiments were performed in a quiet, magnetically shielded room. The participants sat in a chair and watched a silent movie on a screen placed at a distance of 1.5 m in the front throughout the experiment. The left or right MN was randomly stimulated. For MN stimulation of a given side, there were three sound conditions (left, right, and absent) for both electrical and auditory stimulations, thus making a total of six conditions. The MN and auditory stimulations were randomly presented with an even probability with a trial–trial interval of 2000 ms. Analysis began at 100 ms before to 1500 ms after the onset of MN stimulation. A total of at least 100 artifact-free epochs were averaged for each condition. The number of epochs for all conditions and participants was 103.94 ± 2.96, and on average ranged from 100 to 112. The number of epochs did not differ significantly across the conditions (F_5, 66_ = 0.70; p = 0.62).

### Analysis

The N20 m latency for each pulse was analyzed using the source-strength waveform of S1. Dipole analyzes were performed using the Brain Electrical Source Analysis software package (NeuroScan, Mclean, VA). The MEG waveforms of the three conditions of the left MN stimulation were first combined, and a dipole analysis was performed at approximately the peak of N20 m induced by the first electrical pulse. The goodness of fit (GOF) of all participants by the 1-dipole model was over 70% (86.2 ± 7.3 and 85.2 ± 9.0% on the average for left and right MN stimulation, respectively). The same procedure was then applied to the right MN stimulation. To remove the auditory evoked cortical responses, dipoles for the auditory response were included in the dipole model. The MEG waveforms for the two left sound conditions (left MN + left sound and right MN + left sound) were averaged, and dipoles in the auditory cortex on both sides were obtained (Lt-sound dipole). The same procedure was performed for the sound on the right (Rt-sound dipole). Once the dipoles for S1 and bilateral auditory cortex were established, we applied dipole models to the MEG waveforms according to the stimulus combination: 1-dipole model (S1) for the MN condition, 3-dipole model (S1 + Lt-sound dipole) for the MN + Lt-sound condition, and 3-dipole model (S1 + Rt-sound dipole) for the MN + Rt-sound condition. Furthermore, we checked whether the presence of auditory dipoles affected the fit of the S1 dipole. The GOF of the 3-dipole model (S1 + Lt-sound dipole) was higher than that of the 1-dipole model by 0.98% ± 0.89% and 0.46% ± 0.50% for the left and right MN stimulations, respectively. However, the difference was not statistically significant for any participant (p > 0.39)^48^, suggesting that the presence of auditory dipoles did not affect the dipole fit for S1. After establishing the dipole models, the location and orientation of each cortical source was fixed, with variable strength at every sampling point. Using the source-strength waveforms (Fig. 1B) for each stimulus condition, the N20 m latency for each electrical pulse was measured and compared across the different conditions by using two-way repeated measure ANOVA, with sound and hemisphere as independent variables for each electrical pulse in the train. To compare the differences between the conditions, post-hoc multiple comparisons were performed using Bonferroni adjusted *t*-tests. All statistical analyzes were performed with the level of significance set at 0.05.

### Preliminary experiments

Prior to the main experiment, we performed three preliminary experiments. Preliminary Experiments 1 and 2 were performed to determine the stimulation paradigm and the results of the main experiment, and Preliminary Experiment 3 was related to anticipation effects. All preliminary experiments were performed with four of the 12 participants in the main experiment.

In the Preliminary Experiment 1, a pure tone of 25 ms was presented to the left and right side at 25, 50, 75, 100, 125, and 150 ms before single left MN stimulation. Thirteen conditions, including the control condition of single left MN stimulation on its own, were randomly presented (Supplementary Fig. 3A). The procedures for recording and analyzing were similar to those used for the main experiment. There was no change in the N20 m latency under any condition (Supplementary Fig. 3B). We considered the possibility that there was no room for the N20 m latency elicited by a single pulse to be shortened. Therefore, we decided to use MN stimulation of a pulse train in the main experiment, as there was a gradual increase in the N20 m latency during repetitive stimulation as described in the Results.

In the Preliminary Experiment 2, we investigated the methods of the analysis for removing auditory evoked cortical responses. One method focused on the way the auditory response was removed by dipoles for the auditory response, whereas the other method focused on the way the auditory response on its own was subtracted from the multisensory response as suggested by previous studies^61,83^. A pure tone of 25 ms was randomly presented to the left and right side at 1000 ms during left MN stimulation or absence of MN stimulation. The procedures for recording and analyzing were similar to those used in the main experiment. Our results indicated that sounds significantly shortened the N20 m latency at 1050 ms (0.25 ms on average; p = 0.007; paired *t*-test) and 1100 ms (0.44 ms; p = 0.006) in the former analysis and at 1050 ms (0.28 ms on average, p = 0.007) and 1100 ms (0.38 ms, p = 0.005) in the latter analysis (Supplementary Fig. 4). No significant difference was observed between the former and latter analyzes at 1050 ms (p = 0.35) or 1100 ms (p = 0.17). To shorten the recording time, we therefore decided to use the former analysis in the main experiment.

To evaluate the influence of anticipation effects, we performed Preliminary Experiment 3. A pure tone of 25 ms was randomly presented to the left and right side at 800 and 1000 ms during the left MN stimulation. Procedures used for recordings and analyzes were similar to those used in the main experiment. The sound at 800 ms made the N20 m latency faster at 850 ms (0.25 ms on average; p = 0.02; paired *t*-test) and 900 ms (0.34 ms; p = 0.03), but not at 1050 ms (−0.03 ms; p = 0.68) or 1100 ms (0.00 ms; p = 1.00). The sound at 1000 ms shortened the N20 m latency at 1050 ms (0.34 ms; p = 0.04) and 1100 ms (0.53 ms; p = 0.01), but not at 850 ms (−0.09 ms; p = 0.28) or 900 ms (−0.18 ms; p = 0.05) (Supplementary Fig. 5). The results suggested that the acceleration of the sound could not be explained by anticipation.

## Electronic supplementary material

Dataset 1
