# Supplementary material for: Effect of acceleration of auditory inputs on the primary somatosensory cortex in humans
Source: Sci Rep. 2018 Aug 27;8:12883. doi: 10.1038/s41598-018-31319-3 (PMC6110726; doi:10.1038/s41598-018-31319-3)
Supplement: Supplementary file 1 — Dataset 1 [file 41598_2018_31319_MOESM1_ESM.docx]

Effect of acceleration of auditory inputs on the primary somatosensory cortex in humans

Shunsuke Sugiyama^1*^, Nobuyuki Takeuchi^2^, Koji Inui^3, 4^, Makoto Nishihara^5^, Toshiki Shioiri^1^.

^1^Department of Psychiatry and Psychotherapy, Gifu University Graduate School of Medicine, Gifu, Japan; ^2^Depatment of Psychiatry, Aichi Medical University, Nagakute, Japan; ^3^Institute for Developmental Research, Aichi Human Service Center, Kasugai, Japan; ^4^Department of Integrative Physiology, National Institute for Physiological Sciences, Okazaki, Japan; ^5^Multidisciplinary Pain Center, Aichi Medical University, Nagakute, Japan

*Corresponding author

Shunsuke Sugiyama, MD

Department of Psychiatry and Psychotherapy

Gifu University Graduate School of Medicine

Gifu 501-1194, Japan

TEL: 058-230-6000

FAX: 058-230-6266

E-mail: s0450032@yahoo.co.jp

**Supplementary Figure 1.**

**
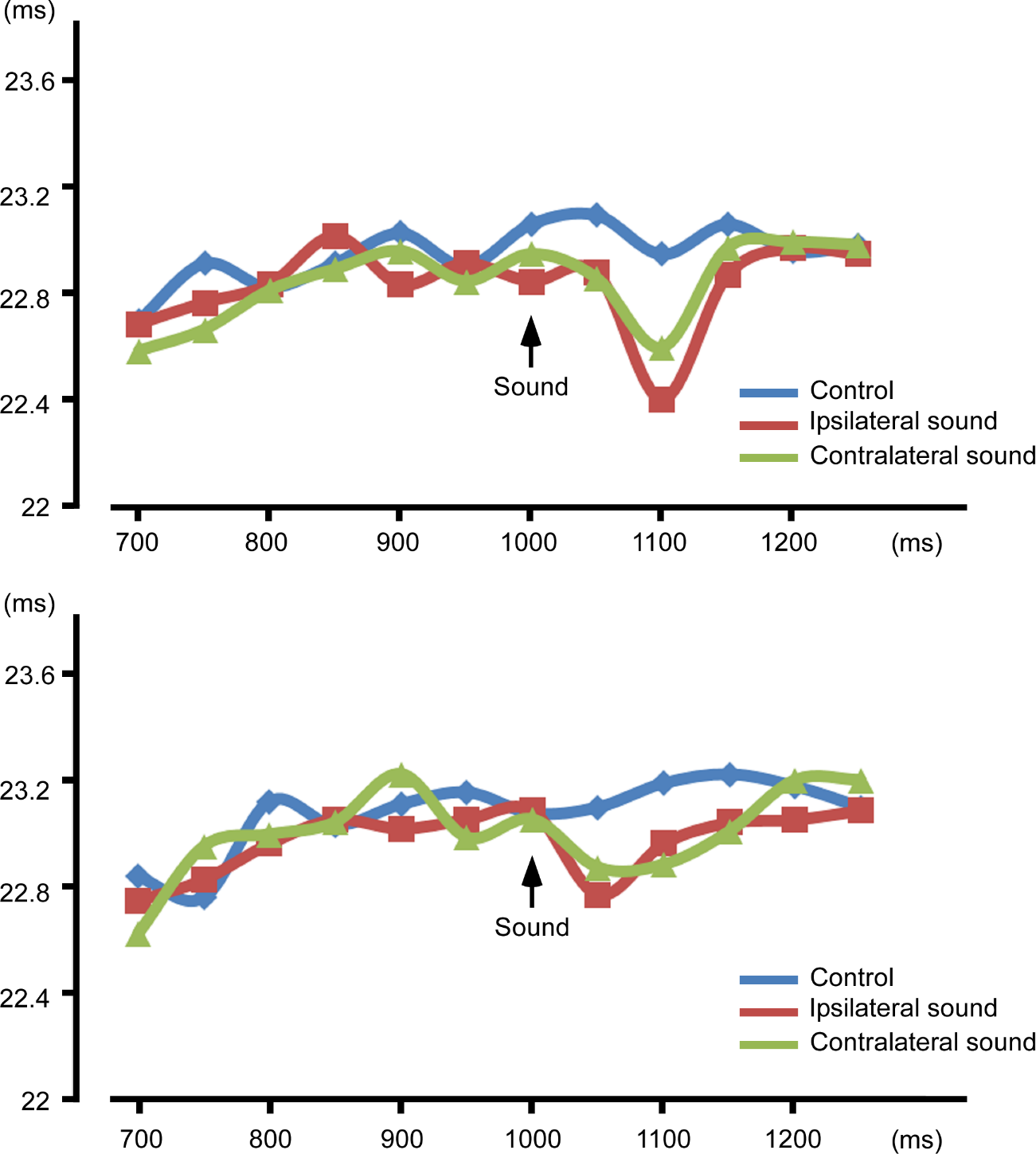
**

**Supplementary Figure 1.** **Comparison of the sound effects on the N20 m latency between the first and second halves of the recording.** The mean peak N20 m latency for each stimulus for the first (top) and second (bottom) halves of the main experiment.

**Supplementary Figure 2.**

**
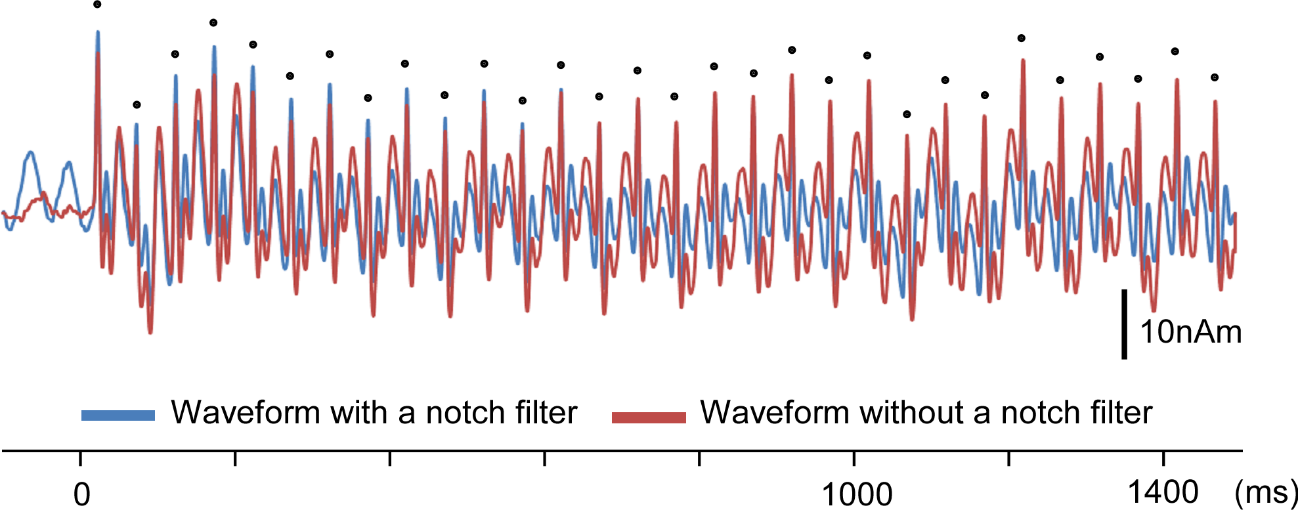
**

**Supplementary Figure 2. Impact of a notch filter on N20 m.** Grand-averaged source strength waveforms of dipoles in area 3b with and without a notch filter of 17.5–22.5 Hz for the left median nerve + left sound condition in Preliminary Experiment 2. Dots, N20 m for each pulse.

**Supplementary Figure 3.**

**
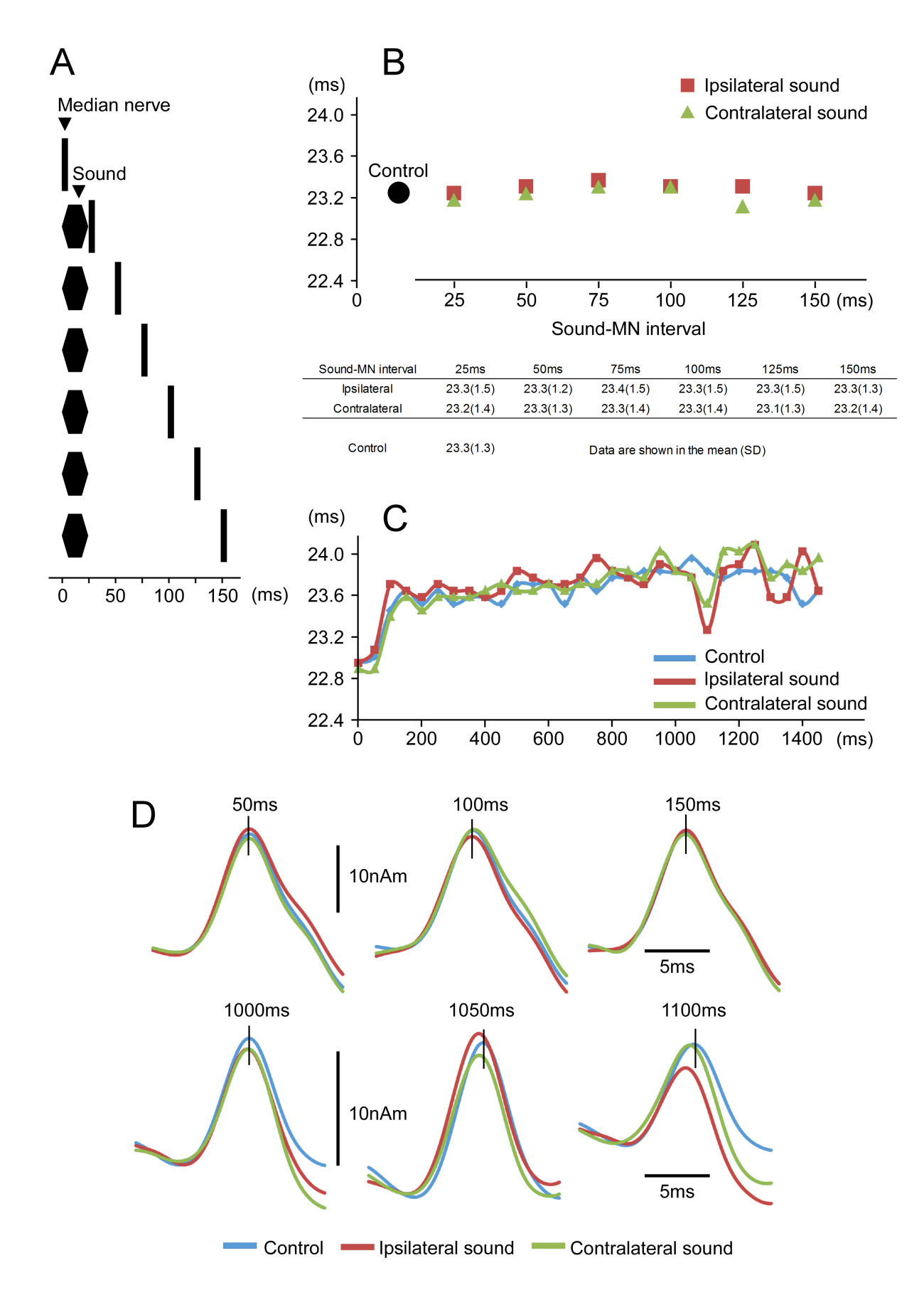
**

**Supplementary Figure 3. Lack of effects of a preceding sound on N20 m after a single pulse.** Data for N20 m after a single pulse to the left median nerve (MN) obtained from four subjects in preliminary Experiment 1. For comparison, the primary experimental results for these four subjects are simulataneously shown. A, stimulation paradigm; B, the mean peak N20 m latency for each condition. The *x*-axis indicates the conditioning-test interval, e.g., 25 ms indicates the N20 m response with the sound 25 ms before the electrical pulse; C, the mean peak N20 m latency for each stimulus for four subjects in the main experiment; and D, grand-averaged N20 m waveforms across four subjects with (50, 100, and 150 ms) and without sound in the preliminary (top) and main (bottom) experiments. Vertical bars, the mean peak N20 m latency for the control condition. Note the absence of a sound effect on the N20 m latency following a single pulse despite evident speeding effects in the main experiment.

**Supplementary Figure 4.**

**
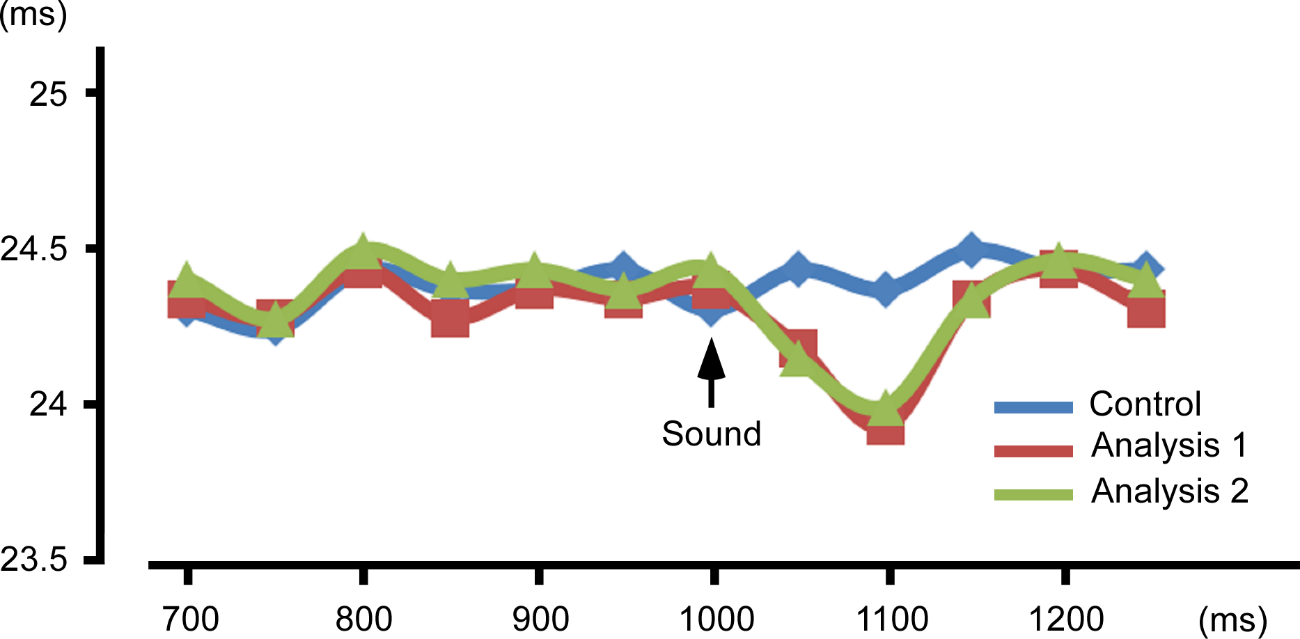
**

**Supplementary Figure 4. Two methods for removing auditory responses.** The mean peak latency of N20 m for each stimulus obtained from four subjects in Preliminary Experiment 2. Results obtained following two different methods for removing auditory evoked responses are shown. In Analysis 1, the auditory response was removed by dipoles for the auditory response, and the N20 m latency was assessed using the residual MEG waveforms as in the main experiment. In Analysis 2, the auditory alone MEG response was subtracted from the multisensory MEG response, and the latency was measured using the subtracted MEG waveforms.

**Supplementary Figure 5.**

**
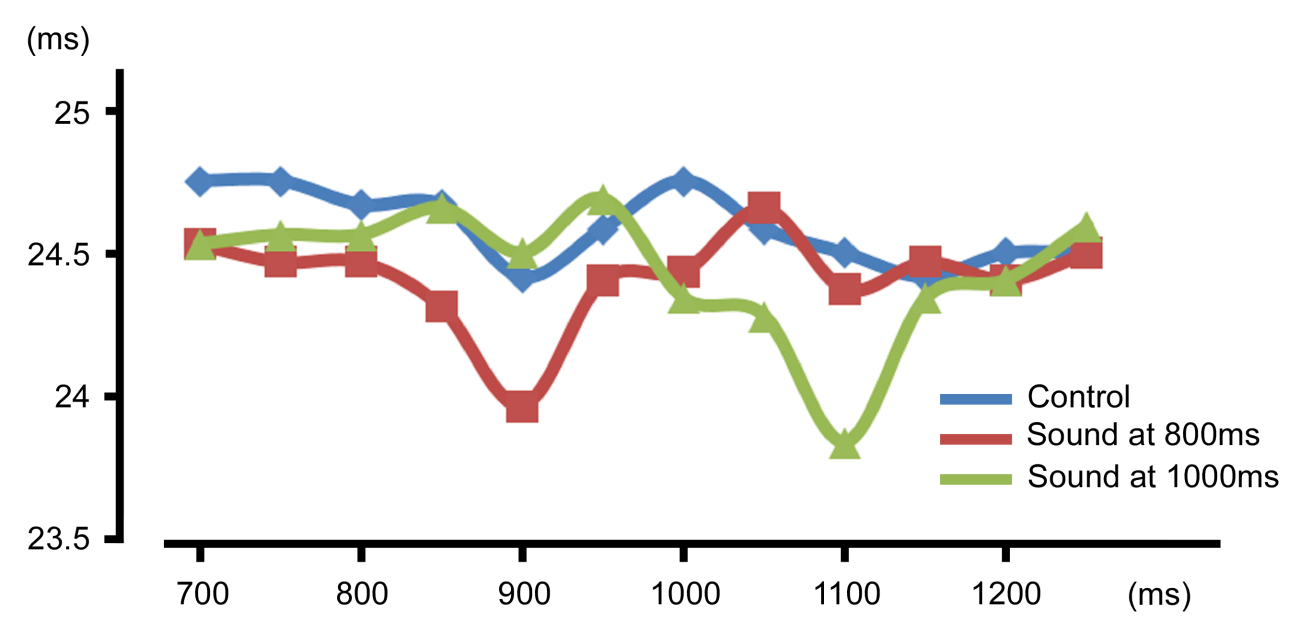
**

**Supplementary Figure 5. Effects of sounds presented at different timings.** The mean peak latency of N20 m for each stimulus obtained from four subjects in Preliminary Experiment 3.

| **Supplementary Table 1. The latencies of N20 m.** | | | | | |  |  |
| --- | --- | --- | --- | --- | --- | --- | --- |
|  |  |  |  |  |  |  |  |
| Latency | Lt median nerve | | |  | Rt median nerve | | |
| (ms) | Control | Lt sound | Rt sound |  | Control | Lt sound | Rt sound |
| 0.0 | 22.3(1.2) | 22.4(1.1) | 22.3(1.3) |  | 22.5(1.1) | 22.5(1.0) | 22.6(1.2) |
| 50.0 | 22.4(1.4) | 22.4(1.4) | 22.4(1.4) |  | 22.2(1.1) | 22.2(1.1) | 22.1(1.0) |
| 100.0 | 22.6(1.5) | 22.8(1.5) | 22.6(1.4) |  | 22.7(1.3) | 22.6(1.1) | 22.6(1.0) |
| 150.0 | 22.6(1.6) | 22.6(1.6) | 22.7(1.7) |  | 22.6(1.3) | 22.5(1.2) | 22.5(1.3) |
| 200.0 | 22.5(1.4) | 22.5(1.4) | 22.5(1.4) |  | 22.5(1.2) | 22.4(1.2) | 22.4(1.2) |
| 250.0 | 22.5(1.5) | 22.6(1.5) | 22.5(1.6) |  | 22.4(1.3) | 22.5(1.2) | 22.5(1.3) |
| 300.0 | 22.4(1.4) | 22.6(1.4) | 22.5(1.4) |  | 22.6(1.3) | 22.6(1.2) | 22.6(1.3) |
| 350.0 | 22.5(1.5) | 22.6(1.3) | 22.6(1.5) |  | 22.7(1.2) | 22.5(1.2) | 22.6(1.3) |
| 400.0 | 22.5(1.4) | 22.6(1.4) | 22.6(1.5) |  | 22.6(1.3) | 22.6(1.3) | 22.5(1.3) |
| 450.0 | 22.6(1.4) | 22.7(1.4) | 22.7(1.4) |  | 22.7(1.3) | 22.8(1.3) | 22.6(1.3) |
| 500.0 | 22.7(1.4) | 22.8(1.4) | 22.7(1.4) |  | 22.8(1.4) | 22.6(1.4) | 22.6(1.4) |
| 550.0 | 22.6(1.5) | 22.8(1.4) | 22.7(1.4) |  | 22.8(1.4) | 22.8(1.3) | 22.7(1.3) |
| 600.0 | 22.7(1.5) | 22.8(1.4) | 22.7(1.4) |  | 22.8(1.2) | 22.7(1.4) | 22.8(1.3) |
| 650.0 | 22.6(1.5) | 22.8(1.4) | 22.7(1.4) |  | 22.8(1.4) | 22.9(1.4) | 22.9(1.3) |
| 700.0 | 22.7(1.5) | 22.8(1.5) | 22.8(1.4) |  | 22.9(1.3) | 22.8(1.4) | 22.8(1.4) |
| 750.0 | 22.7(1.5) | 22.9(1.4) | 22.8(1.4) |  | 22.9(1.4) | 22.9(1.4) | 22.9(1.4) |
| 800.0 | 22.8(1.5) | 22.9(1.4) | 22.8(1.5) |  | 22.9(1.3) | 23.0(1.3) | 22.9(1.4) |
| 850.0 | 22.8(1.5) | 22.9(1.5) | 22.9(1.4) |  | 23.0(1.4) | 23.0(1.3) | 23.0(1.3) |
| 900.0 | 22.8(1.5) | 22.8(1.4) | 22.8(1.5) |  | 23.1(1.4) | 22.9(1.4) | 22.8(1.5) |
| 950.0 | 22.8(1.5) | 22.9(1.4) | 22.9(1.5) |  | 23.1(1.4) | 22.9(1.4) | 22.9(1.4) |
| 1000.0 | 22.8(1.5) | 22.9(1.4) | 22.8(1.5) |  | 22.9(1.4) | 23.0(1.5) | 22.9(1.3) |
| 1050.0 | 22.8(1.5) | 22.8(1.4) | 22.7(1.5) |  | 23.1(1.3) | 22.8(1.4) | 22.7(1.4) |
| 1100.0 | 22.8(1.6) | 22.6(1.4) | 22.6(1.5) |  | 23.0(1.4) | 22.8(1.3) | 22.6(1.4) |
| 1150.0 | 22.8(1.6) | 22.9(1.5) | 23.0(1.5) |  | 23.0(1.4) | 23.0(1.4) | 22.9(1.4) |
| 1200.0 | 22.8(1.6) | 22.9(1.5) | 23.0(1.4) |  | 23.0(1.4) | 22.9(1.4) | 22.9(1.3) |
| 1250.0 | 22.8(1.6) | 23.0(1.5) | 23.0(1.5) |  | 23.0(1.4) | 23.1(1.4) | 22.9(1.6) |
| 1300.0 | 22.8(1.5) | 22.8(1.5) | 22.8(1.5) |  | 22.9(1.4) | 23.0(1.4) | 22.9(1.3) |
| 1350.0 | 22.9(1.6) | 22.8(1.5) | 22.8(1.6) |  | 23.0(1.4) | 23.0(1.3) | 23.0(1.5) |
| 1400.0 | 22.7(1.6) | 22.9(1.5) | 22.9(1.5) |  | 22.9(1.3) | 22.9(1.3) | 22.9(1.5) |
| 1450.0 | 22.7(1.6) | 22.8(1.6) | 22.9(1.5) |  | 22.9(1.4) | 23.0(1.4) | 23.0(1.4) |
| Data are shown in the mean (SD) | | | |  |  |  |  |
